# Infected RBC flag/parameter provided by Mindray BC-6800 haematology analyzer aid the diagnosis of malaria

**DOI:** 10.1186/s12936-019-2890-z

**Published:** 2019-07-31

**Authors:** Yi Sun, Daijun Xiang, Chen Chen, Shang He, Huan Qi, Chengbin Wang

**Affiliations:** 1 Department of Clinical Laboratory, Chinese People’s Liberation Army General Hospital, Beijing, 100853 China; 2 Algorithm and Clinical Research Department, Haematology, IVD, Shenzhen Mindray Bio-Medical Electronics Co., Ltd, Shenzhen, 518057 China

**Keywords:** Malaria, Parasitaemia, Automatic alert, Infected RBC, Light microscopy, BC-6800 analyzer

## Abstract

**Background:**

The Mindray BC-6800 haematology analyzer (BC-6800) provides a dedicated flag ‘Infected RBC’ (InR) and the number of InR (InR#)/the permillage of InR (InR‰) in routine blood testing as a screening tool for malaria in endemic areas. This study sought to evaluate the effectiveness of the BC-6800 flag parameter for aiding the diagnosis of malaria.

**Methods:**

A total of 181 samples were tested using the Mindray BC-6800 haematology analyzer, including 117 malaria-infected samples collected from Yunnan, China, and 64 samples from healthy controls. Microscopy examination was conducted as reference when stained thick blood film revealed the presence of malaria parasites identified as *Plasmodium vivax* and *Plasmodium falciparum*. The receiver operating characteristic (ROC) curve analysis was developed using Analyse-it v4.92.3. The Kappa value was determined to evaluate the agreement between BC-6800 and light microscopy.

**Results:**

The sensitivity of InR‰ generated by BC-6800 for *P. vivax* and *P. falciparum* was 88.3 and 24.1%, respectively; specificity of InR‰ for malaria parasites was 84.3 and 84.3%, respectively; positive predictive value and negative predictive value was 89.4 and 82.7% for *P. vivax,* and 52.8 and 60.3% for *P. falciparum*. There was a strong correlation between ΔWBC and InR‰ (R^2^ = 0.9731 for *P. vivax* and R^2^ = 0.9757 for *P. falciparum*). There was also a significant correlation between parasitaemia and InR# in *P. vivax*-infected samples (R^2^ = 0.734). InR# was evaluated using ROC curve analysis, the area under the ROC curve is 0.95 with a 95% confidence interval of 0.926 to 0.974, and the cut-off value is 0.01 × 10^9^/L for *P. vivax*. However, the ring stage and the early trophozoite stage of *Plasmodium* cannot be detected easily on BC-6800, possibly because of the small size and low nucleic acid content of these stages.

**Conclusions:**

The findings suggest that the flag ‘InR’ and the parameters ‘InR#/InR‰’ provided by the BC-6800 haematology analyzer could be used to screen for malaria in a clinical setting.

## Background

Malaria is a vector-borne infectious disease that continues to have high morbidity and mortality globally [1]. The primary clinical presentation of malaria is fever or flu-like symptoms or a history of fever and flu-like symptoms. Diagnosis based only on clinical symptoms has very low specificity [2] as there is no combination of symptoms that reliably distinguishes malaria from other causes of fever or flu.

Light microscopy, malaria nucleic acid amplification (PCR) test and malaria rapid diagnostic tests (RDTs) are used for parasitological diagnosis of malaria. Malaria PCR is not commonly used due to its high cost; RDTs are now more common, but not yet the regular test in non-endemic areas; microscopic examination of stained blood films remains the standard and most commonly used diagnosis method. Although this is the standard method, it is labour intensive and requires a high level of expertise to scan thick blood films for the presence of malaria parasites and thin blood films to determine the type of malaria [3, 4].

Malaria is prevalent in parts of Southeast Asia, South Asia, Africa and South America and, therefore, there is a demand for malaria screening in these areas [5–7]. Haematology analysis technology has improved considerably in the last 70 years, particularly with the introduction of automated haematology analyzers. Modern analyzers are capable of processing hundreds of samples per hour by using flow cytometry, and such techniques could aid in the diagnosis of malaria [8–11]. A recent study introduced a novel analyzer, which was able to detect infected red blood cells (iRBCs) in blood samples from mice infected with rodent malarial parasites [12] and determine the developmental stage of cultured *Plasmodium falciparum* [13]. Although this research is promising, most analyzers using numeric and graphical data to detect malaria fail to move beyond the research phase and are not clinically applicable due to the careful monitoring required to detect malaria parasites. A more reliable and accessible detection method for malaria is a dedicated alert message/flag that is incorporated into routine complete blood count (CBC) analysis when a positive case is detected. This could support earlier detection and potentially reduce adverse outcomes related to malaria infection.

For clinicians without extensive technical experience or expertise, the BC-6800 automated haematology analyzer (Mindray, Shenzhen, China) could be used to detect malaria parasites and to calculate parasitaemia in blood samples through cytometry analysis. The BC-6800 analyzer provides CBC parameters, reticulocyte and its fractions, and nucleated red blood cells (NRBC) value. It also provide a dedicated flag for ‘InR’ and ‘InR#/InR‰’ parameters. These are research use only parameters roughly equivalent to the number of InR (InR#)/the permillage of InR (InR‰) for the malaria iRBCs in a sample [14]. BC-6800 light scatter and fluorescence three-dimensional analysis technology (SF Cube) detects ‘iRBCs’ using signals generated by side-scattered light (SC, representing the internal cell structure and its contents), forward-scattered light (FS, indicating iRBC size), and side fluorescent light (FL, corresponding to DNA content). The flag is generated without the use of any special reagents.

The present study aimed to evaluate the utility of the infected RBC flag and parameters InR#/InR‰ in routine blood testing as a malaria screening tool in endemic areas. Furthermore, a comparison study was carried out for the flag information of CBC parameters between control group and the malaria group with *P. vivax* and *P. falciparum.*

## Methods

### Samples and technical principles

EDTA anti-coagulated blood samples were collected from Tengchong, Yunnan Province (an endemic area in the border region of China and Myanmar) between May and August in 2016 according to physicians’ request. Some 117 malaria-infected samples were analysed, including 96 from *P. vivax*-infected patients (aged 3–69 years; 80 men and 16 women) and 21 from *P. falciparum*-infected patients (aged 6–60 years; 14 men and 7 women). Sixty-four healthy subjects from a non-endemic area in China were collected as the control group (aged 8–70 years; 47 men and 17 women). Samples were classified as malaria positive only when microscopy examination of stained thick blood film revealed the presence of at least one of the four malaria parasite forms: ring form, trophozoite, schizont and/or gametocytes. All blood samples were examined using both light microscopy and BC-6800 haematology analyzer using the manufacturer recommended reagents, calibrator and controls. Analyzer performance was monitored daily using three levels of quality control material.

The BC-6800 haematology analyzer used sheath flow impedance, laser scatter and SF Cube analysis technology. The SF Cube analysis technology is three-dimensional using information from laser light scatter at two angles and fluorescent signals for cell differentiation and counting [15]. In the BC-6800 differentiating (DIFF) channel, the fluorescent staining technology was adopted after the sample was mixed with DIFF lyse. For samples infected with malaria, RBC and white blood cell (WBC) sub-populations were differentiated by their size and complexity using lysing. Due to the different content of nucleic acid in WBC sub-populations, the volume of fluorescent dye staining the nucleic acid substances was different: the low-angle light scatter reflects cell size, the high-angle light scatter reflects intracellular granularity, and the intensity of fluorescent signal reflects the degree to which the cell is stained. Since multiple proteins play a role in generating increased rigidity of *Plasmodium*-infected erythrocytes, that would result in resistance to lysis [16, 17]. Through three-dimensional signal analysis of the cells processed with lyse, the DIFF channel differentiates the sub-populations, including lymphocytes, monocytes, neutrophils, *Plasmodium*-infected RBC, and eosinophils, as well as identifies and flags abnormal cells such as immature granulocytes, abnormal lymphocytes and blast cells.

### Comparison with light microscopy and quality control

Thin and thick blood films were prepared for staining and parasite investigation. Blood films were sent to Tengchong Laboratory in a standard slide box for staining with 3% Giemsa and microscopic examination [18]. Parasitaemia was determined from thick blood films by counting the number of parasites per 200 WBCs. Thick blood films were classified as positive if one or more malaria parasites were observed and negative if no parasites were observed after examining at least 100 ‘oil-lens’ fields (i.e. at a magnification of ×1000). Thin smears were further examined after parasites were seen in the thick smears in order to measure parasitaemia and identify the species of malaria parasites. All the blood smears were examined using a CX21 light microscope (Olympus, Tokyo, Japan).

The malaria microscopist was blinded to BC-6800 results. Similarly, healthy controls were blinded to microscopy results. In order to assure malaria form quality of the microscopic examinations, the entire positive and 10% of the negative slides were sent to a senior malaria microscopist and re-examined at the Tengchong Laboratory. An experienced reader was assigned to re-examine the discrepant slides.

### Ethical consideration

This study obtained ethical approval from the ethics committee of the General Hospital of the People’s Liberation Army. Individual informed consent was obtained from adults and from the parents or guardians of children under 18 years old when they presented in hospital. In addition, verbal assents were given to minors.

### Data analysis

Data entry was performed using Microsoft Office Excel. Statistical analysis was performed using SPSS 22.0 (SPSS Inc, Chicago, IL, USA) analyzer Microsoft tool box. It was compared on the blood cell parameters and suspect flags between control group and iRBC group in BC-6800 analyzer. Pearson’s correlation or Spearman’s rank correlation was also used to determine the association between control group and malaria group (*P. vivax* group and *P. falciparum* group). Sensitivity, specificity and predictive values for the detection of different *Plasmodium* species were calculated and compared the *P. vivax* group and *P. falciparum* group. InR# representing the number of ‘iRBC’ was evaluated using ROC curve analysis, reporting the area under the curve (AUC) and its confidence interval (CI). ROC analysis was completed using Analyse-it v4.92.3. The Kappa value was calculated to evaluate the agreement between BC-6800 and light microscopy. A *P* value less than 0.05 was considered statistically significant.

## Results

### A special cluster showed in BC-6800 scattergram with malaria-infected samples

**Fig. 1:**
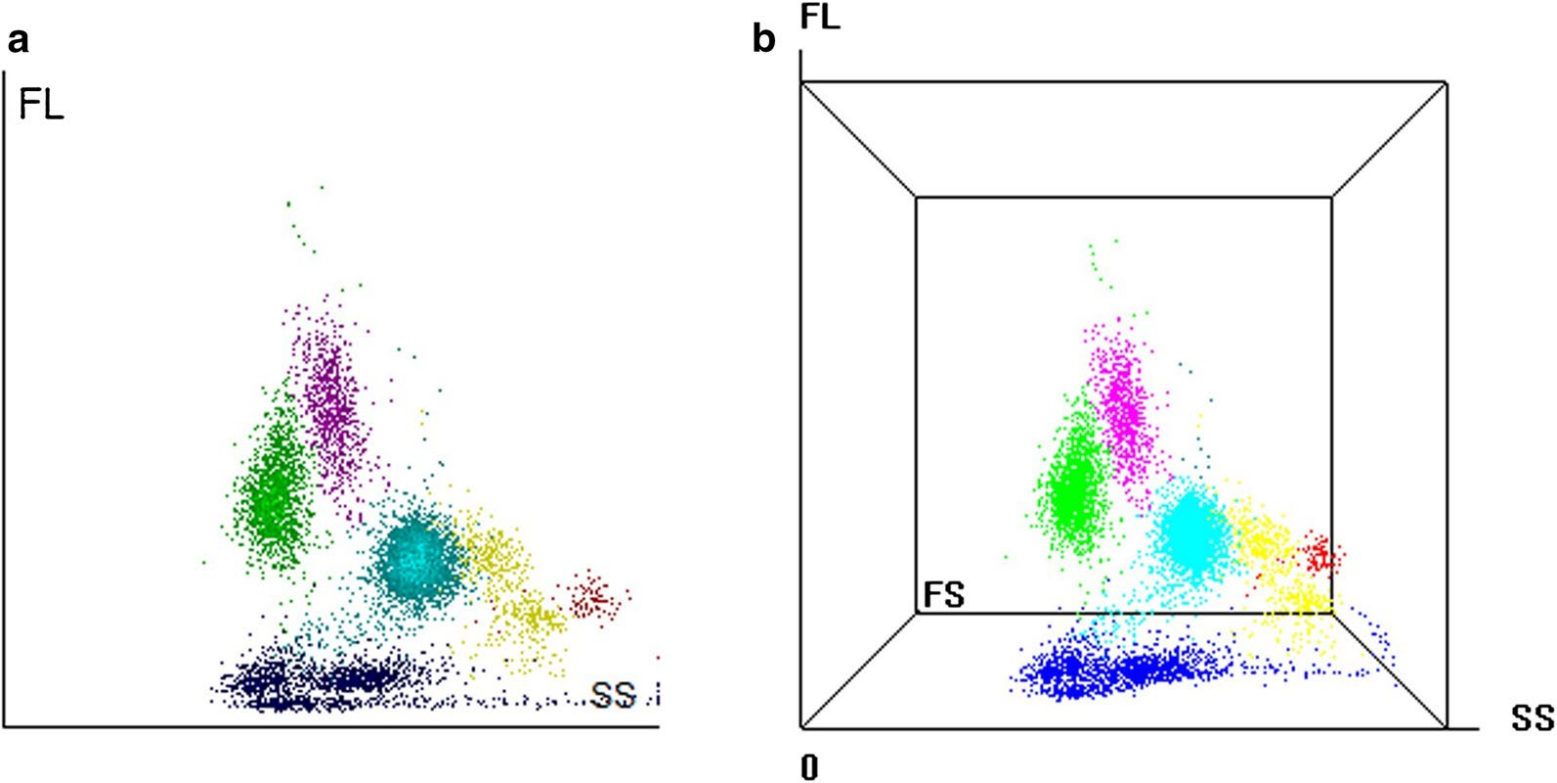
White blood cell scattergrams generated by the Mindray BC-6800 haematology analyzer. **a** An example of the gates lymphocyte, monocyte, neutrophil, infected RBC (yellow spots) and eosinophil in a representative patient with malaria; **b** the distinct cluster of malaria parasites (yellow spots) in three-dimensional analysis scattergrams

BC-6800 WBC DIFF plot displayed a distinct cluster for malaria parasites (Fig. 1a, yellow spots); SF Cube scatter plot permits clearer view of infected cell cluster (Fig. 1b, yellow spots). These findings suggested that samples with malaria parasites, especially schizont and/or gametocytes, show a distinct cluster and unique location on SF Cube.

### Performance of InR by BC-6800 in comparison to the reference light microscopy

The sensitivity and specificity of light microscopy for *P. vivax* and *P. falciparum* infection were compared with that of InR‰ by BC-6800. The sensitivity of the InR‰ by BC-6800 for *P. vivax* and *P. falciparum* was 88.3 and 24.1%, respectively. The specificity of the InR‰ by BC-6800 for *P. vivax* and *P. falciparum* was 84.3 and 84.3%, respectively. Details of these results can be found in Table 1.

**Table 1:** Performance of InR‰ by BC-6800 identified in comparison to the reference light microscopy

	*P. vivax* (95% CI)	*P. falciparum* (95% CI)
Sensitivity	88.3% (83.73–93.77%)	24.1% (19.43–40.42%)
Specificity	84.3 (76–90.55%)	84.3% (76–90.55%)
Positive likelihood ratio	5.69 (3.66–8.82)	1.85 (1.06–3.22)
Negative likelihood ratio	0.12 (0.08–0.20)	0.84 (0.71–0.99)
Disease prevalence	60% (53.89–65.89%)	42.25 (35.07–49.67%)
Positive predicitve value	89.4% (83.73–93.77%)	52.8 (40.89–72.96%)
Negative predicitve value	82.7% (76–90.55)	60.3 (53.54–69.78%)
Total consistent rate	86.7%	58.9%
Kappa value	0.738	0.143

**Fig. 2:**
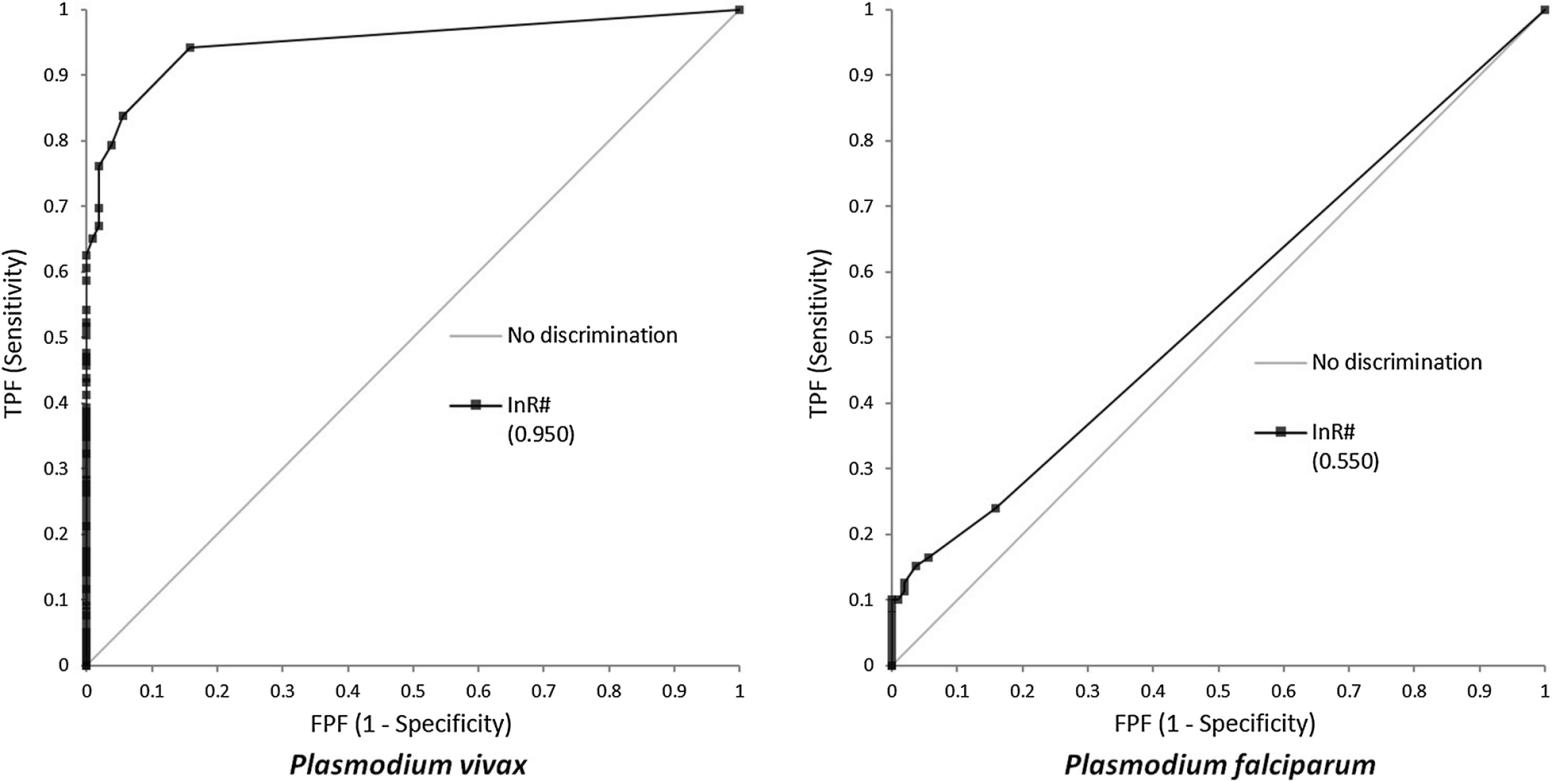
InR# was evaluated by the ROC curve on *Plasmodium vivax* and *Plasmodium falciparum*

InR# was evaluated using ROC curve analysis. In the *P. vivax* group, AUC is 0.95, with a 95% CI of 0.926 to 0.974 and Youden indices of 0.726. The best cut-off is 0.01 × 10^9^/L. However, in the *P. falciparum* group, area under ROC curve is only 0.55, meaning it is not amenable to screen *P. falciparum.* The ROC curves are shown in Fig. 2.

### The difference of InR and InR‰ in various infection density groups

As shown in Table 2, the results presented that the infection densities in microscopy (χ^2^ = 16.230, P < 0.001) in various InR groups were unequal, and the difference was statistically significant. In addition, these results displayed that the infection density in InR group II and group III were higher than that in group I.

**Table 2:** The difference of infection density in different groups (*Plasmodium vivax*) [M (P25, P75)]

The number of InR	Infection density in microscope (10^9^/L)	*χ* ^2^	*P*
Group I	1388 (932, 2040)	16.23	< 0.001
Group II	2575 (1217, 4485)*		
Group III	2957 (2105, 5201)*		

I: The range of InR# was [0, 0.1); II: The range of InR# was [0.1, 2); III: The range of InR# was ≥ 2

* Compared to group I, *P* < 0.05

### Blood cell parameters and suspect flags between control group and malaria groups in BC-6800 analyzer

#### WBC parameters and suspect flags between control group and malaria group

**Fig. 3:**
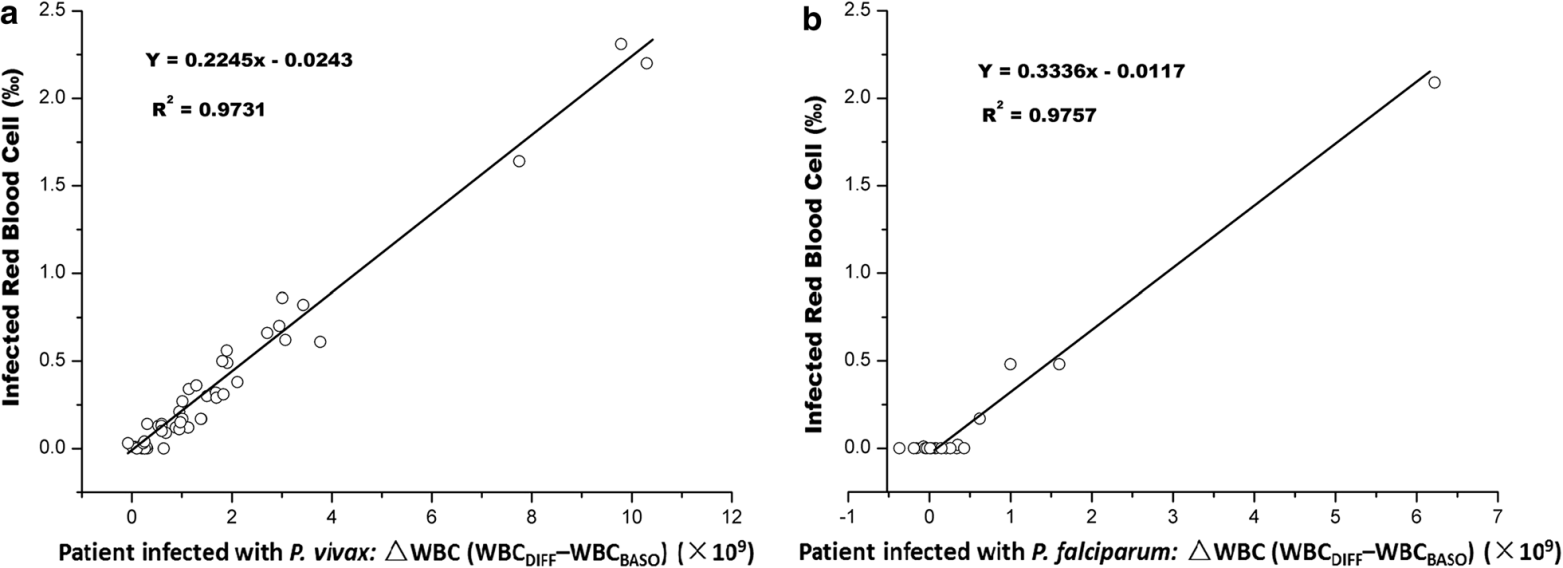
The correlation between the ‘Infected RBC’ (InR‰) and ΔWBC (WBC_DIFF_–WBC_BASE_). **a** The correlation between the InR‰ and ΔWBC in *P. vivax*; **b** the correlation between the InR‰ and ΔWBC in *P. falciparum*

**Fig. 4:**
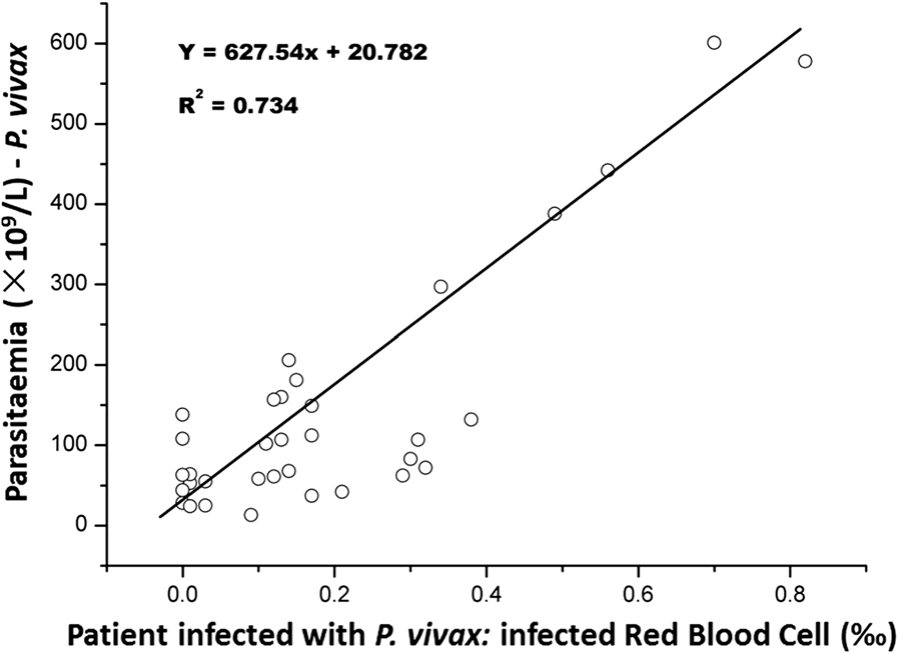
The correlation between the ‘Infected RBC’ (InR‰) and *P. vivax*

Automated WBC counts and suspect flags from BC-6800 haematology analyzer are shown in Table 3. The correlation between the count of InR and cell blood count was analysed in the *P. vivax*-infected patient group and in the *P. falciparum*-infected group. The result showed that there was a clear relationship between ΔWBC (WBC_DIFF_–WBC_BASO_) and InR‰ (*P. vivax* group R^2^ = 0.973). (WBC_DIFF_ is the number of WBC counting in the DIFF channel with mild lyse, and WBC_BASO_ is the number of WBC counting in the BASO channel with severe membrane destruction in the Mindray BC-6800 haematology analyzer). A better relationship was present between ΔWBC and InR‰ in the *P. falciparum* group, however, the InR flags were only flagged in 5 of all 21 *P. falciparum* patients by the BC-6800 analyzer (Fig. 3). The correlation between the number of InR (‰) and *P. vivax* was R^2^ = 0.734 (Fig. 4).

**Table 3:** Blood cell parameters and suspect flags between control group and malaria groups in BC-6800 analyzer

Parameters	Control group (n = 64)	Malaria group (n = 117)		*p*_1_	*p*_2_
		*P. vivax* (n = 96)	*P. falciparum* (n = 21)		
WBC [DIFF] count (× 10^9^/L)	6.94 (2.75 to 22.65)	6.46 (2.13 to 21.0)	6.14 (2.55 to 12.44)	0.04	0.03
WBC [BASO] count (× 10^9^/L)	6.90 (2.74 to 22.84)	5.42 (1.06 to 11.31)	5.70 (2.29 to 9.76)	0.02	0.03
WBC [DIFF]–WBC [BASO] (× 10^9^/L)	0.04 (− 5.8 to 16.12)	1.75 (− 0.37 to 1.6)	0.5 (− 0.67 to 2.06)	0.01	0.01
RBC (10^12^/L)	4.93 (3.13 to 7.06)	4.66 (1.64 to 6.82)	4.54 (2.49 to 6.53)	0.72	0.68
HGB (g/L)	136.78 (97 to 208)	128.54 (52 to 209)	125.15 (62 to 165)	0.83	0.81
RDW-CV (%)	13.45 (11.8 to 17.3)	14.55(11.2 to 32.4)	14.55 (12 to 18.2)	0.66	0.66
RET (%)	0.98 (0.33 to 2.67)	1.53 (0.43 to 12.47)	2.01 (0.52 to 7.58)	0.01	0.01
IRF (%)	4.44 (0 to 17.5)	9.90 (0.9 to 24.2)	6.80 (0.9 to 25.1)	0.01	0.02
LFR (%)	95.56 (82.5 to 100)	90.10 (56.1 to 99.1)	93.21 (74.9 to 99.1)	0.04	0.06
MFR (%)	4.28 (0 to 13.4)	8.11 (0.9 to 24.2)	6.23 (0.9 to 19.3)	0.03	0.04
HFR (%)	0.16 (0 to 4.2)	1.79 (0 to 22.1)	0.57 (0 to 5.8)	0.01	0.02
Anaemia (%)	11 (17.18%)	27 (28.13%)	6 (28.57%)	0.03	0.03
PLT count (× 10^10^/L)	193 (62 to 355)	108 (8 to 289)	149 (53 to 323)	0.01	0.03
Malaria flag/parameters					
InR flag	0	85 (88.5%)	5 (23.80%)	0.01	0.01
InR#					
InR‰					

*P*_1_, the *P* value compared *P. vivax* group to control group; *P*_2_, the *P* value compared *P. falciparum* group to control group; InR, parasite infected red blood cell; InR#, the number of infected red blood cell; InR‰, the permillage of infected red blood cell. RBC, red blood cell; HGB, haemoglobin; RDW, red blood cell volume distribution width; RET, reticulocyte; IRF, immature reticulocyte fraction; LFR, low fluorescence ratio; MFR, middle fluorescence ratio; HFR, high fluorescence ratio; PLT, platelet count; InR, parasite infected red blood cell; InR#, the number of infected red blood cell; InR‰, the permillage of infected red blood cell

#### RBC parameters and suspect flags between control group and malaria group

Automated RBC counts and suspect flags from BC-6800 haematology analyzer are shown in Table 3. There was no significant difference in RBC, HGB and red blood cell volume distribution width (RDW) between the malaria group and control group (P > 0.05), but a significant difference in reticulocyte parameters was observed between *P. vivax/P. falciparum* patient group and the control group (P < 0.01).

#### PLT parameters and suspect flags between control group and malaria group

Automated platelets (PLT) counts and suspect flags from the BC-6800 haematology analyzer are shown in Table 3. There was significant difference in PLT count between *P. vivax/P. falciparum* patient groups and control group (P < 0.05).

## Discussion

The BC-6800 haematology analyzer uses SF-Cube technology to achieve three-dimensional cell analysis for WBC, reticulocyte (RET) and NRBC with clinically relevant suspect flags [19, 20]. It provides a dedicated flag named ‘infected RBC?’ and parameters ‘InR#/InR‰’ to represent the possible presence of *Plasmodium* parasites and the causative agent of malaria infection. In this study, the parameter ‘infected RBC’ was used to analyse the diagnosis performance in patients with malaria, as well as routine parameters and suspect flags.

The diagnostic performance of the InR flag for the diagnosis of *Plasmodium* was evaluated. Samples with malaria parasites show a distinct cluster and unique location on three-dimensional cell plots. The characteristics of the cluster are dependent on the species of *Plasmodium* as well as the size and number of the parasite present. The sensitivity and specificity for detecting *P. vivax* was not significant for *P. falciparum*. This may be a result of early trophozoites of *P. falciparum* that developed over 10 h in the peripheral blood concealed in the microvasculature, sinusoids or other slow blood flow before being developed into late trophozoites and schizonts [21, 22]. Trophozoites and schizonts of *P. falciparum* are rarely seen in the peripheral blood of infected patients. The ring and the gametocyte stage are the most commonly seen in a peripheral blood smear. However, the ring and the early trophozoites of *Plasmodium* cannot be detected easily on BC-6800, the main reason possibly being the small size and low nucleic acid content.

InR flag being triggered by BC-6800 should meet two conditions. First, the malaria-infected specimens should be collected during the intra-erythrocytic phase *Plasmodium*. Second, the number of intra-erythrocytic phase *Plasmodium* should surpass a certain threshold [23]. Some *P. vivax* specimens were tested as false negatives because they did not meet these two conditions. This may be because the majority of samples among the *P. vivax* group are trophozoite and ring form, which are too small to be detected easily. Both microscopy and malaria RDT are very effective at identifying ring infections. In addition to its poor sensitivity for detecting *P. falciparum*, this study suggested the shortcoming should be improved in BC-6800 haematological analyzer. At present, this is the major limitation of broader use for *P. falciparum* in particular. The machine could be more effective for identifying *P. falciparum* in future.

Several studies report that DIFF scattergram suspect flags can provide assistance in the diagnosis of malaria in *P. vivax*-infected patients [10, 24]. Results from the present study support this finding in the iRBC group. Research shows that high eosinophil count is found in the presence of *P. vivax* infection, and was observed in up to 39% *P. vivax*-infected patients [25]. In this study, results found that pseudo-eosinophilia was observed in only a small number of samples (*P. vivax* 6.25% and *P. falciparum* 14.29%). In *P. vivax* and *P. falciparum*-infected patients, results found that WBC count was higher in the DIFF channel (mild lyse) compared to the WBC/BASO channel (severe membrane destruction). A strong correlation was found between ∆WBC (WBC_DIFF_–WBC_BASO_) and the number of iRBC (R^2^ = 0.973) for *P. vivax*-infected patients (Fig. 3). A correlation was also found between parasitaemia and the number of iRBC for *P. vivax*-infected patients (R^2^ = 0.734; Fig. 4). This makes it possible to estimate the amount of *P. vivax* after samples are tested using the BC-6800.

Anaemia a common symptom of malaria and is associated with dyserythropoiesis and ineffective erythropoiesis [26]. The BC-6800 haematology analyzer provides additional parameters, including the immature platelet fraction (IPF) and immature reticulocyte fraction (IRF), both important in clinical markers for thrombocytopaenia and anaemia [27–29]. Results from the present study show *P. vivax* or *P. falciparum* parasitaemia are associated with abnormal reticulocyte parameters, such as RET scattergrams and IRF. Results also show platelet-associated flags, including PLT clump, IPF and PLT abnormal in malaria-positive samples were increased compared to the control group. These findings are in accordance with the study of Dubreuil et al. [30].

Except for cost, malaria PCR and RDTs are obviously ahead of the BC-6800 in sensitivity. It is necessary to further improve the sensitivity, especially for the ring infections, in the apparatus. In this study, the associations were limited between the haematological parameters and the species of infection considering patient age, symptoms and prior treatment.

## Conclusions

A dedicated ‘Infected RBC’ flag on a CBC result print-out provides an objective record and a trigger for further examination if malaria is suspected. Establishing a work protocol to further examine all flagged samples and use thick blood film microscopy and/or malaria RDTs to confirm or rule out malaria may reduce therapeutic intervention time and improve patient care outcomes. In malaria-endemic zones, ‘Infected RBC’ flag could serve as a rapid decision support tool when screening for malaria.

## Data Availability

The data used and/or analysed during the current study are available from the testing of blood samples collected from area mentioned above.

## Abbreviations

iRBC: infected red blood cell
CBC: complete blood count
NRBC: nucleated red blood cells
InR: infected RBC
RBC: red blood cell
WBC: white blood cell
ROC: receiver operating characteristic
AUC: area under the curve
CI: confidence interval
RDW: red blood cell volume distribution width
DIFF: differentiating
PLT: platelets
RET: reticulocyte
PPV: positive predictive value
NPV: negative predictive value
malaria RDT: malaria rapid diagnostic test
SC: side scattered light
FS: forward scattered light
FL: side fluorescent light
